# Specific microbial gene abundances and soil parameters contribute to C, N, and greenhouse gas process rates after land use change in Southern Amazonian Soils

**DOI:** 10.3389/fmicb.2015.01057

**Published:** 2015-10-06

**Authors:** Daniel R. Lammel, Brigitte J. Feigl, Carlos C. Cerri, Klaus Nüsslein

**Affiliations:** ^1^Centro de Energia Nuclear na Agricultura, University of São PauloPiracicaba, Brazil; ^2^Department of Microbiology, University of MassachusettsAmherst, MA, USA

**Keywords:** tropical rainforest, biogeochemical cycles, microbial indicators, qPCR, soil processes

## Abstract

Ecological processes regulating soil carbon (C) and nitrogen (N) cycles are still poorly understood, especially in the world’s largest agricultural frontier in Southern Amazonia. We analyzed soil parameters in samples from pristine rainforest and after land use change to pasture and crop fields, and correlated them with abundance of functional and phylogenetic marker genes (*amoA*, *nirK*, *nirS*, *norB*, *nosZ*, *nifH*, *mcrA*, *pmoA*, and 16S/18S rRNA). Additionally, we integrated these parameters using path analysis and multiple regressions. Following forest removal, concentrations of soil C and N declined, and pH and nutrient levels increased, which influenced microbial abundances and biogeochemical processes. A seasonal trend was observed, suggesting that abundances of microbial groups were restored to near native levels after the dry winter fallow. Integration of the marker gene abundances with soil parameters using path analysis and multiple regressions provided good predictions of biogeochemical processes, such as the fluxes of NO_3_, N_2_O, CO_2_, and CH_4_. In the wet season, agricultural soil showed the highest abundance of nitrifiers (*amoA*) and Archaea, however, forest soils showed the highest abundances of denitrifiers (*nirK*, *nosZ*) and high N, which correlated with increased N_2_O emissions. Methanogens (*mcrA*) and methanotrophs (*pmoA*) were more abundant in forest soil, but methane flux was highest in pasture sites, which was related to soil compaction. Rather than analyzing direct correlations, the data integration using multivariate tools provided a better overview of biogeochemical processes. Overall, in the wet season, land use change from forest to agriculture reduced the abundance of different functional microbial groups related to the soil C and N cycles; integrating the gene abundance data and soil parameters provided a comprehensive overview of these interactions. Path analysis and multiple regressions addressed the need for more comprehensive approaches to improve our mechanistic understanding of biogeochemical cycles.

## Introduction

Biogeochemical processes regulating the carbon (C) and nitrogen (N) cycles are still poorly understood, mainly because of the lack of adequate microbial indicators. Recent studies have shown the potential for using cultivation-independent measures, and that the quantified marker genes are good indicators of the associated biogeochemical processes in the C and N cycles (Morales et al., 2010; Petersen et al., 2012; Levy-Booth et al., 2014; Rocca et al., 2014). This approach includes the analysis of key protein-encoding genes related to these processes and their correlation and/or fit into models with standard chemical and environmental parameters (e.g., C and N forms, pH, temperature, and water content).

Several genes have been used as molecular markers to determine the abundance of a respective microbial group in soils (e.g., 16S rRNA genes; Petersen et al., 2012), and biogeochemical processes have been quantified using specific genes related to indicative enzymatic steps in the C or N cycles (Supplementary Table S1). In the N cycle, the *nifH* gene (encoding nitrogenase, that reduces N_2_ from the atmosphere to ammonium) is related to N-fixation; *amoA* (ammonia monooxygenase) is related to nitrification; and *nirK* (copper nitrite reductase), *nirS* (iron nitrite reductase), *norB* (nitric oxide reductase), and *nosZ* (nitrous oxide reductase) are related to denitrification (Levy-Booth et al., 2014). In the methane cycle, the *mcrA* gene (methyl coenzyme M reductase) is linked exclusively to methanogens; and the *pmoA* gene (methane oxygenase) to methane-utilizing prokaryotes (methanotrophs), among several other examples (Rocca et al., 2014).

An example of the application of this approach is the study of Petersen et al. (2012), who quantified the *amoA*, *nirK*, *nirS*, and *nosZ* genes to estimate nitrification and denitrification potential in Alaskan soils, and determined them suitable indicators of these processes. Other examples are given in a review by Levy-Booth et al. (2014), in which the authors reviewed studies that had employed these and other genes as indicators of transformations within the N cycle. Recently, Rocca et al. (2014) performed a meta-analysis showing that gene quantification is still a novel approach and is constantly improving. In a comparative analysis of 59 selected studies, even though these studies were from different groups and used independent methods, the authors found significant correlations of gene quantification with C and N cycle processes (*r* = 0.30, *P* < 0.001). Based on this information, we argue that the use of gene quantification goes beyond the improvement of predictive models, and has the potential to provide information about the dynamics and ecology of the organisms directly related to these processes.

Most models attempting to predict C, N, and greenhouse gas (GHG) dynamics in soils rarely acknowledge detailed microbiological parameters (Bouwman, 1998; Rocca et al., 2014). Although the models mention the microbial processes involved, they rarely present data about the specific microbial species or functional groups which mediate these processes. Microbial information is commonly limited to general indicators such as microbial biomass, and the focus is on microbial activity alone. However, microbial activity is a consequence of the concentration of microbial enzymes and the environmental conditions that regulate their activity (e.g., substrate, temperature, available water, and O_2_; Burns et al., 2013). Since soil is a complex environment for biochemical studies, the improvement of techniques for soil enzymes has been a constant challenge (Burns et al., 2013), and consequently, the physico-chemical environmental parameters which regulate enzyme activity are solely used to explain activity (e.g., “hole-in-the pipe” model; Bouwman, 1998). Methods are required that also include the abundance and ecology of the respective microorganisms that mediate the underlying process, to provide a better scientific understanding of these microorganisms and to potentially improve model accuracy (Morales et al., 2010; Petersen et al., 2012; Levy-Booth et al., 2014; Rocca et al., 2014).

Currently the best approach to identify and quantify specific microbial groups and/or enzymes related to processes of the C and N cycle is the use of cultivation independent molecular tools, e.g., the quantification of marker genes that encode the process-related enzymes (Morales et al., 2010; Petersen et al., 2012). The mRNA transcripts are not a valid parameter, since mRNA is a transitory and unstable molecule, and RNA transcripts can vary from a dozen copies to a thousand copies per cell, changing numbers within minutes depending on environmental conditions (Deutscher, 2006; Rocca et al., 2014). Accurate protein detection and quantification is still a challenge in soils (Levy-Booth et al., 2014). The quantification of genes based on genomic DNA is a powerful approach to detect the potential (dependent on other factors such as substrate availability, water and O_2_) of a soil to perform the associated process (Levy-Booth et al., 2014; Rocca et al., 2014). Furthermore, the quantification of such genes enables an approximate quantification of microorganisms that belong to that functional group (the copy numbers of these genes are generally one to two copies per cell). Moreover, this strategy enables the study of the microbial organisms that perform these processes, information which is usually missing from biogeochemical approaches that focus only on environmental physico-chemical parameters, and incorporates important information about the ecology of these organisms in soils (Petersen et al., 2012; Levy-Booth et al., 2014; Rocca et al., 2014).

Here we applied the quantification of specific functional marker genes to provide important insights into consequences of land use change from pristine rainforest to agricultural uses in Southern Amazonia, the largest agriculture frontier worldwide. In Brazil, agriculture is of great social, economic, and environmental importance. Because of the growing domestic and global demand for agricultural products, the country has continually expanded its agricultural activities in the past decades. Today Brazil is the second biggest soybean and livestock producer in the world (Food Agriculture Organization [FAO], 2014), and these agricultural areas are constantly expanding from their origins in the south further north in the direction of the Amazonian rainforest. Within Brazil the state of Mato Grosso in Southern Amazonia ranks as the biggest producer of soybeans, and contains the largest area covered by pasture in Brazil. This region represents the most dynamic agricultural expansion worldwide, where tropical rainforests have been converted into pasture and crop lands, but little is known about the impacts of these land use changes on soil biogeochemical cycles (Galford et al., 2010, 2011).

Following deforestation, not only plant biomass but also the stocks of total C and N in soil can become mobilized and be partially emitted in the form of the greenhouse gasses CO_2_, CH_4_, and N_2_O (Cerri et al., 2006). Some studies conducted in Southern Amazonia have evaluated the impact of land use change on soil organic matter (SOM) and GHG fluxes (Neill et al., 1997; Carvalho et al., 2010; Maia et al., 2010, Galford et al., 2011), and found that losses of soil C and N stocks are greatly modified by agricultural management. Conventional tillage, overgrazing, and low chemical fertility can lead to a significant loss of soil C, but specific agricultural conservation practices, such as no-tillage, can increase SOM accumulation in these tropical soils (Carvalho et al., 2010; Maia et al., 2010). Although C, N, and GHG cycles have been studied in parts of Southern Amazonia, little is known about the microorganisms responsible for these processes in this region (Carvalho et al., 2010).

The objective of this study was to analyze *in situ* how the abundance of microbial functional groups related to the soil C and N cycles shifted in response to land use change from rainforest to agricultural use in Southern Amazonia. Furthermore, we investigated whether these changes were correlated with changes in soil C and N content, or with soil GHG fluxes (CO_2_, CH_4_, and N_2_O). We compared a pristine rainforest with adjacent pasture and soybean sites established on the same soil approximately 25 years ago following deforestation. For further comparison, we included another agricultural site within the same ecotype, which has soybeans established only 2 years after forest conversion, enabling the detection of short-term effects on biogeochemical cycles in this region.

The primary hypothesis for this investigation was that the effects of land use change on the abundance of microbial functional groups are related to the total soil C and N concentrations, and to GHG fluxes in Southern Amazonia. A secondary hypothesis states that the abundances of microbial marker genes are appropriate indicators to model processes within the respective biogeochemical cycles.

## Materials and Methods

### Site Survey and Soil Sampling

This study was conducted on three farms nearby the municipality of Sinop, Mato Grosso State, Brazil, one of the most important agricultural regions in Southern Amazonia (Galford et al., 2010). Typical farms with similar edapho-climatic conditions and known land use history were chosen (Supplementary Table S2). The forest on the chosen farms was identified as a semideciduous mesophytic forest, with Amazonian species affinity (Ackerly et al., 1989), and the soils were classified as Red Oxisol with clay texture. In the past, the same forest type was removed at a large scale from adjacent areas (same soil type) and converted to agriculture. The climate is megathermic, with wet summers and short, dry winters, or ‘Am’ by the Köppen-Geiser climate classification. The average temperature is 24.1°C, and the average annual precipitation is 2171 mm (Vourlitis et al., 2008). Old established soybean fields in this region are typically cultivated using a no-till system with double cropping with corn or sorghum, while new soybean fields after deforestation are typically cultivated using conventional tillage (Carvalho et al., 2007). Both soybean fields receive annual fertilization, periodic liming (Ca and Mg carbonates), and pesticides; in contrast, pastures rarely receive annual fertilization or liming (Lammel et al., 2015). The land use types sampled and their geographic coordinates were: 12°05′22″S, 55°28′24″W (25 years soybean field), 12°05′26″S, 55°28′35″W (pasture), 12°05′34″S, 55°28′43″W (adjacent forest), and 11°44′49″S 56°15′14″W (2 year soybean field and adjacent forest; Supplementary Table S2).

Soil samples were surveyed during two different time periods. The first sampling was in the 1st week of November 2010, at the beginning of the wet season, and just prior to soybean seeding. Samples were taken a second time in January 2011, in the middle of the wet period, and immediately after soybean flowering, termed stage R3 (Fehr et al., 1971). The characteristic dry period of Southern Amazonia falls between June and September, therefore sampling efforts were focused on the beginning (November) and the intermediate wet season (January), assuming higher microbial activity during this time because of higher soil moisture, and higher average temperature. This period also coincides with the soybean growth stages of seedling (October–November) and maximum growth (January). For the two soil surveys DNA was extracted and gene quantification performed.

Soil was sampled from two (November 2010) or six replicated plots (January 2011) for each land use type. Within each replicated plot five soil samples were surveyed, and a total of 10 and 30 samples were studied per land use type at each sampling time, respectively. Replicated plots were located from dozens to 100 m away from each other and were characterized by the same topography and soil type (Red Oxisol). This is the predominant soil type in this agricultural region, and to reduce potential bias caused by soil variation we only surveyed samples of this soil type (Maia et al., 2010). Land use history and soil profile analyses confirmed that the agricultural sites had been converted from the same adjacent forest and soil types. In each of the replicated plots, soil samples were collected in a cross shaped design with four samples in the cardinal directions located 20 m equidistant to the fifth sample in a central position.

The soil survey in the middle of the wet season (January 2011) was performed together with a soil gas flux survey, with the aim of correlating soil and microbial parameters with the quantification of GHG fluxes. For this, we collected soils from six replicated plots for each land use type within a 1 week period (January 15–19), as described above. Within that week, two replicate sites were surveyed for each land use type in three subsequent sampling events spaced 2 days apart, January 15, 17, and 19. Thus, a total of 30 samples were analyzed for each land use type, originating from six replicated plots, each with five sub-samples. At the same time two replicated plots were sampled from the 2 year-old soybean sites, yielding an additional 10 samples (surveyed on January 16).

After removing the litter layer, soil was sampled from 0 to 17 cm using sterile PVC tubes. Soil cores were immediately packaged in sampling bags and placed on ice, to be frozen later on the day of sampling at -20°C (Lammel et al., 2015). One week later, the frozen soil cores were used to extract DNA, nitrate, and ammonium. All samples were individually processed to avoid cross contamination, and all equipment was disinfected with 80% ethanol prior to sampling. In the survey during the wet season, soil cores were collected immediately after GHG sampling inside the ring base of the gas sampling chamber to ensure that the soil cores corresponded to their respective *in situ* gas fluxes (for details on gas sampling see below).

### Chemical Analysis

Soil samples were processed according to the standard methods for Brazilian tropical soils used by the Agronomic Institute of Campinas (IAC; Cantarella et al., 1998). The pH values were measured in 0.01 M CaCl_2_; exchangeable cations (K^+^, Ca^2+^_,_ and Mg^2+^) and available P were extracted using ion exchange resins; the trace elements Cu, Fe, Mn, and Zn were extracted by diethylenetriaminepentaacetic acid and triethanolamine with a pH of 7.3, and B was thermally extracted in water (Cantarella et al., 1998; Carvalho et al., 2007). Ammonium (NH_4_^+^) and nitrate (NO_3_^-^) were extracted by adding 4 g frozen soil to 40 ml of 1 M KCl; the suspension was agitated for one h, and filtered through quantitative filter paper at a pore size of 8 μm (Lammel et al., 2015). The extract was analyzed in a flow injection analysis system. NH_4_^+^ concentration was determined by conductivity detection, and NO_3_^-^ was reduced in a Cd column, and measured colorimetrically (Cerri et al., 2006). Total C and total N were determined using a C/N Analyzer CN-2000 (LECO, St. Joseph, MI, USA; Carvalho et al., 2010).

### Quantitative PCR Analysis

Total DNA was extracted from 0.25 g of soil using the Power Lyzer Power Soil DNA Isolation Kit (MoBio, Solana Beach, CA, USA), according to the manufacturer’s instructions, and stored at -20°C. For each of the two (November) or six (January) replicated plots, DNA extraction was performed independently for each of the five sub-samples (soil cores). The resulting five DNA extracts were pooled at equal volumes, resulting in one DNA sample for each replicated plot (*n* = 2 or 6), and used as templates for qPCR.

Quantitative PCR was performed in an Opticon2 device (Bio-Rad, Berkeley, CA, USA) in 96-well plates. All analyses were performed at least in duplicate. Each reaction was composed of 1 μl of extracted template DNA, 0.2 μl of KlenTaq DNA polymerase, 1 × KlenTaq buffer (DNA Technology, St. Louis, MO, USA), 0.5 μg of T4 Gene 32 Protein, to both increase template detection sensitivity and suppress humic acid inhibition (Tebbe and Vahjen, 1993; Kermekchiev et al., 2009), 1 × EvaGreen (Biotium, Hayward, CA, USA), 0.25 mM of dNTPs, primer pairs as described (Supplementary Table S1), and molecular grade water to a final volume of 20 μl (Rotthauwe et al., 1997; Hallin and Lindgren, 1999; van Elsas et al., 2000; Throbäck et al., 2004; Rösch and Bothe, 2005; Yu et al., 2005; Henry and Bru, 2006; Dandie et al., 2007; Mcdonald et al., 2008; Steinberg and Regan, 2008; Claesson et al., 2010). Standards for quantification were prepared from PCR amplified genes from environmental DNA using each primer set, and dilutions were employed as qPCR standards using the method described by Hou et al. (2010). The thermocycler was programmed with an initial denaturation step at 95°C for 5 min, followed by 40 cycles of 95°C denaturation for 15 s, annealing (temperature specified in **Table 1**) for 30 s, and an extension at 68°C for 30 s, followed by a final extension cycle of 5 min at 68°C. All primer pairs were chosen according to the literature available in 2010, and amplification protocols were optimized for our laboratory conditions (for details, refer to **Table 1**). Melting curve analyses were performed from 68 to 95°C, and standard curves had *R*^2^ higher than 0.99. The amplified fragments were also confirmed in agarose gel for specificity and size (Petersen et al., 2012). No significant inhibition was observed when standards were compared to soil extracts spiked with the addition of standards. Amplification efficiencies were 84 ± 9%, a value that is in the range of similar studies (Meyer et al., 2013; Jones et al., 2014).

**Table 1:** Selected soil attributes, and gene abundances and gas fluxes in the surveyed areas in the wet season (see the full data set in Supplementary Table S3).

Attribute	Unit	Forest	Soybean 2y	Soybean 25y	Pasture
**Soil Chemistry**			
C	mg.g^-1^	2.9a^1^	2.6b	2.3c	2.5
N	mg.g^-1^	0.17a	0.14c	0.14c	0.15b
NH_4_	μg.g^-1^	4.7a	0.7c	2.2b	2.6b
NO_3_	μg.g^-1^	3.0a	0.8b	0.7b	0.9b
pH	–	3.9c	5.1a	5.0a	4.6b
Ca	mmol_c_.dm^-3^	2.0c	25.0a	27.7a	11.7b
Mg	mmol_c_.dm^-3^	2.0c	19.7a	6.3b	5.7b
P	mg.dm^-3^	4.3b	16.0a	13.7a	3.3b
Cu	mg.dm^-3^	0.2b	0.7a	0.4ab	0.1b
**Marker genes**			
*16S Archaea*	copies.g soil^-1^	8.8E + 06b	7.4E + 06c	1.3E + 07a	9.1E + 06b
*16S Bacteria*	copies.g soil^-1^	2.7E + 09a	2.1E + 08c	3.5E + 08c	1.1E + 09b
*18S Fungi*	copies.g soil^-1^	6.0E + 06a	1.6E + 05b	1.9E + 05b	4.9E + 06a
**N-cycle genes**			
*nifH*	copies.g soil^-1^	2.1E + 07a	2.9E + 06c	5.1E + 06c	1.5E + 07b
*amoA Archaea*	copies.g soil^-1^	5.8E + 05d	1.5E + 06c	6.0E + 06a	4.0E + 06b
*amoA Bacteria*	copies.g soil^-1^	4.8E + 05a	2.0E + 05b	1.7E + 05b	2.3E + 05b
*norB*	copies.g soil^-1^	2.0E + 06a	5.7E + 05b	1.5E + 06a	2.4E + 06a
*nirK*	copies.g soil^-1^	7.4E + 06a	5.9E + 05c	2.0E + 06c	4.0E + 06b
*nirS*	copies.g soil^-1^	2.2E + 06b	4.5E + 05c	7.6E + 05c	4.6E + 06a
*nosZ*	copies.g soil^-1^	2.3E + 07a	1.7E + 06c	1.9E + 06c	8.8E + 06b
**Methane genes**			
*mcrA*	copies.g soil^-1^	2.1E + 05a	1.4E + 04c	2.3E + 04c	7.2E + 04b
*pmoA*	copies.g soil^-1^	6.7E + 07a	9.8E + 06c	1.0E + 07c	3.1E + 07b
**Gasses**			
CO_2_ Flux	mg C-CO_2_.m^2^ soil. h^-1^	129b	258a	98b	111b
CH_4_ Flux	μg C-CH_4_.m^2^ soil. h^-1^	-7b	-14b	8b	46a
N_2_O Flux	μg N-N_2_O.m^2^ soil. h^-1^	33a	9b	4b	4b

*^1^Values with the same letter in one row are not different by Tukey’s post hoc test (p < 0.05).*

### GHG Analysis

Soil gas fluxes were measured using a two-piece static polyvinyl chloride plastic chamber (Steudler et al., 1989). Five chamber-bases, separated from each other by 20 m, were inserted 2 cm into the soil at each sampling site in a cross design (*n* = 30, consisting of five chambers for each of the six replicated plots). After mounting the gas-tight chamber lid, headspace gas samples of 10 ml were surveyed using nylon syringes at the beginning of each incubation (time 0), and 10 and 20 min thereafter. Individual samples were transferred to glass tubes sealed with air tight stoppers and crimped for storage until analysis. Air chamber temperature, soil temperature at 5 and 10 cm depths, and atmospheric barometric pressure were measured. All gas samples were analyzed immediately after the survey, within a week, using the gas chromatograph GC-2014-GHG (Shimadzu, Kyoto, Japan). The fluxes of each gas were determined by calculating the changing concentration in the chambers as a function of incubation time, and adjusted for pressure, chamber volume, and temperature (Cerri et al., 2006).

### Statistical Analysis

The data were analyzed as a mixed model considering a nested design, where the replicated sites were nested with the surveyed days (*n* = 6 for qPCR data) and individual soil cores (*n* = 30 for all forms of C and N, and GHG fluxes; Pinheiro and Bates, 2000). Data were Box–Cox transformed and submitted to an ANOVA, and when a significant effect was observed, a Tukey *post hoc* test was performed (*P* < 0.05). All tables and graphs are shown as averages of the replicate plots (*n* = 2 for the November 2010 samples, *n* = 6 for the January 2011 samples, with the exception of soybean 2 years where *n* = 2). Pearson correlation and principal components analysis (PCA) were performed to test relationships between the variables, and n-MANOVA to check if the land use types were statistically different (Anderson, 2001). All analyses were run using the software R, and the libraries car, lme, and vegan (R Core Team, 2013).

To further evaluate the fit of the marker gene abundances with environmental parameters in biogeochemical processes, multiple regression, and path analysis were performed. Five models were tested, considering C degradation and CO_2_ flux, nitrification, denitrification, and methane flux (Supplementary Figure S1). Path analyses were performed as described by Petersen et al. (2012), based on the reduction of the full models, and selection of the best models where all paths were statistically significant. Some of our data presented a moderately skewed distribution, thus path models were analyzed using Bollen–Stine bootstraps in the R-library Lavaan (Rosseel, 2012). The general fit of the model is shown, with a desirable *P* > 0.05, Comparative Fit Index (CFI) > 0.93 (0 low fit, 1 best fit), and a Standardized Root Mean Square Residual (SRMR) < 0.08, with the individual paths having *P* < 0.05 (Rosseel, 2012). Multiple regressions were performed for both parametric models and general additive models using smoothing spline within the R-library mgcv, which supports semi and non-parametric distributions (Wood, 2006).

## Results

Land use change from forest to pasture or soybean resulted in several changes in soil physicochemical and microbial parameters. Forest soil was characterized by higher concentrations of total C and N as well as higher available mineral N values (NH_4_^+^ and NO_3_^-^) compared to all agricultural sites (**Table 1**). After conversion to agriculture, pH values and soil nutrient concentrations increased (P, Ca, Mg), most notably in the soybean fields (**Table 1** and Supplementary Table S3). These chemical changes paralleled shifts in abundances and activities of microbial functional groups (**Table 1**).

The initial characterization of gene abundance in soils was made in November, at the beginning of the wet season. No statistically significant differences in gene abundance were observed among land uses in November (Tukey, *P* < 0.05; Supplementary Figure S2), suggesting that the effect of land use change on the microbial community was low. Significant differences in gene abundances were obtained between November and January samples (*P* < 0.05). In January, during the middle of the wet season, the differences between forest and derived sites were evident (**Table 1**, *P* < 0.05), therefore the present study focused on this period. All the results below refer to the January samples (to avoid confusion, results from the November samples are given only in the Supplementary Information).

### Microbial Abundance and Activity

Different land uses were characterized by distinct patterns of microbial abundance. Abundances of Bacteria and Fungi were highest in forest sites, while Archaea dominated old soybean fields (**Table 1**). Bacterial and fungal abundance were negatively correlated with pH, soil bases (Ca, Mg, K), P, and Zn, and positively correlated with total N, NO_3_^-^, CEC, H^+^+Al^+3^, and Fe. In addition, Bacterial abundance was also positively correlated with total C, CEC, B, Fe, Mn, and B (Supplementary Table S4). Microbial activity, represented by CO_2_ flux, was more intense in the new soybean soils, and no difference was observed among the other land use types (**Table 1**).

**FIGURE 1:**
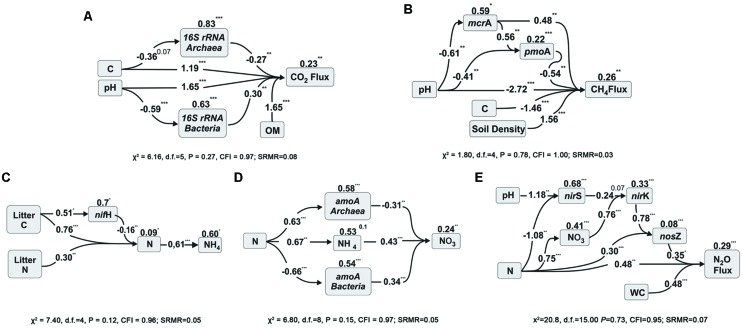
**Path analysis diagrams for microbial gene abundances and CO_2_ fluxes (A); for methane dynamics (B); and for N-cycle related processes, NH_4_^+^ prediction (C), NO_3_^-^ prediction (D) and N_2_O fluxes (E).** The diagrams show the relationships among the selected variables and the influence of soil chemical factors in soils across land use types in Southern Amazonia in the middle of the wet season (for more information please see the Methods section). The numbers listed within arrows are standardized path coefficients (^∗^*P* < 0.05, ^∗∗^*P* < 0.01, ^∗∗∗^*P* < 0.001, or x *P* < x). The numbers on the top of the variable boxes represent unexplained variation (1 – *R*^2^) which represents the effect of unmeasured variables and measurement error. The litter C and N data are from Lammel et al. (2015).

**FIGURE 2:**
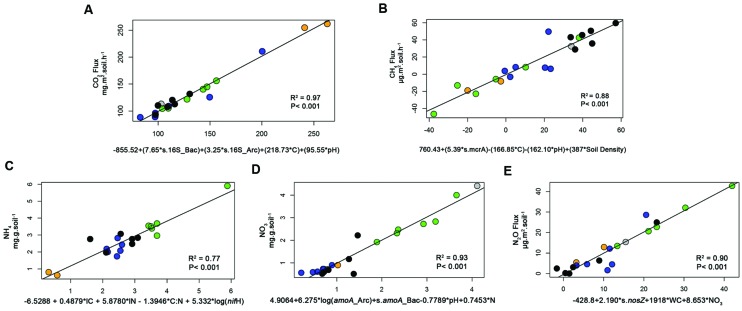
**Linear regression analyses (reporting the *R*^2^ and *P*-value) between selected processes, CO_2_ fluxes (A), CH_4_ fluxes (B), NH_4_^+^ prediction (C), NO_3_^-^ prediction (D), N_2_O fluxes (E), and an equation made with the coefficients from the best multiple regression result for each process (for details please see Methods and the Supplementary Information “Regressions”).** These graphs demonstrate the fit (*R*^2^) of our data in the prediction equations based on the multiple regression analyses. Data are shown for four different land use types, forest (green), pasture (black), soybean 25 years (blue), soybean 2 years (orange squares, and adjacent forest in gray).

To further investigate the interaction among the variables, two techniques were used to test models with these variables and to explain the soil respiration. The first was path analysis, which tested selected combinations of the variables based on an initial full model (Supplementary Figure S1). That initial full model was reduced to a significant model where all the paths were significant (**Figure 1A**). In this path diagram, the soil C negatively influenced Archaeal abundance, the soil pH negatively influenced Bacterial abundance, and all of them in addition to OM influenced the CO_2_ flux. The numbers on the top of each variable boxes represent unexplained variation (1 – *R*^2^), which represents the effect of unmeasured variables and measurement error (Petersen et al., 2012). This means that in this model only 0.23 of the CO_2_ Flux was not explained by this model; and that Archaea and Bacteria contributed a little to the model, with 0.83 and 0.63 of their variance not explained by this model. The other technique used was stepwise multiple regressions, and in this case, there was no guided dependency among variables as that stated in the path analysis (Supplementary Information “Regressions”). Full models were than tested and reduced for models where all the coefficients were statically significant and the best Akaike information criterion (AIC) achieved. The coefficients of the best analysis were then used in a linear regression against the CO_2_ flux, and achieved an *R*^2^ of 0.97 and *P* < 0.001 (**Figure 2A**). Both independent techniques showed that the abundance of 16S genes of Archaea and Bacteria contributed to the explanation of the CO_2_ fluxes from the analyzed samples.

### N-Cycle Dynamics

**FIGURE 3:**
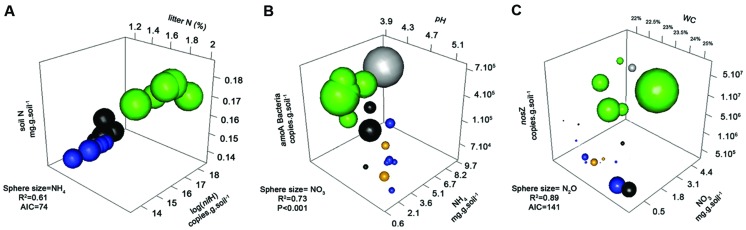
**3-D bubble plots based on univariate data to explain the products NH4+ (A), NO_3_^-^ (B), and N_2_O Flux (C).** The axes are the predictor variables and the sphere sizes the respective products. For each graph, its significance was tested by a regression analysis, reporting its *R*^2^ and significance (*P* or AIC). Data are shown for four different land use types, forest (green), pasture (black), soybean 25 years (blue), soybean 2 years (orange squares, and adjacent forest in gray).

**FIGURE 4:**
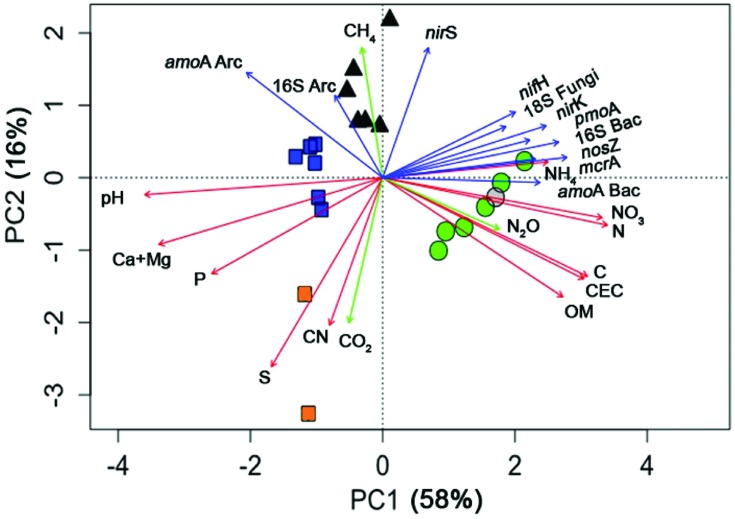
**Principal Components Analysis of land uses (symbols) and soil parameters (vectors) in Southern Amazonia in the middle of the wet season.** Data are shown for four different land use types, forest (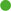), pasture (▲), soybean 25 years (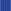), soybean 2 years (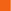, and adjacent forest in a gray circle); and vectors for the soil chemical parameters, microbial genes, and GHG fluxes.

The abundance of N-fixers, based on *nifH* genes, was highest in forest soils, followed by pasture soils. Archaeal ammonium oxidizers (AOAs) were numerically dominant at all sites, and overall abundance (*amoA* Archaea) was highest in the old soybean fields, and lowest in forest soils. Bacterial ammonium oxidizers (AOB) were more abundant in the forest sites (**Table 1**). The abundance of denitrification genes was highest in forest soils, intermediate in pasture, and lowest in soybean fields (**Table 1**). Significant correlations and relationships between related genes (*nirK* and *nosZ*) were found, and also between them and N, NO_3_^-^, NH_4_^+^, and N_2_O (Supplementary Table S4). Interestingly, abundances of *nifH*, *amoA* Bacteria, *nirK*, and *nosZ* genes were correlated between each other (in fact, they are all present in some bacteria, such as *Rhizobium* sp.). Interactions among the variables were further characterized by 3D plots and multivariate techniques (**Figures 3** and **4**).

To explain NH_4_^+^ concentrations in soil, a 3D bubble plot showed the relationships among soil N, N-fixers (*nifH*) and litter N (litter data from Lammel et al., 2015) (**Figure 3A**, a linear-mixed model for this data showed *R*^2^ = 0.61, AIC = 74). These relationships were also demonstrated by a path analysis (**Figure 1C**). The path diagram indicated that litter C, litter N, N-fixers, and soil N were statically significant to explain NH_4_^+^ concentrations, however, the NH_4_^+^ unexplained variation (1 – *R*^2^) in this model was 0.60. Soil N had the highest fit to the path diagram, and had only 0.09 of unexplained variation. The best multiple regression result (Supplementary Information “Regressions”) incorporated soil C:N, litter C, litter N, and *nifH* gene as variables; when their coefficients were used in a linear regression with NH_4_^+^, the *R*^2^ was 0.77 and *P* < 0.001 (**Figure 3C**).

To explain NO_3_^-^ in soil, a 3D bubble plot showed the relationships among soil NH_4_^+^, nitrifiers (*amoA* Bacteria) and pH (**Figure 3B**, a linear model for this data showed *R*^2^ = 0.73, *P* < 0.01). The path diagram indicated that soil N, NH_4_^+^, *amoA* Archaea and *amoA* Bacteria were statically significant to explain the NO_3_^-^ concentrations, and it presented 0.23 of unexplained variation in this model (**Figure 1D**). Soil NH_4_^+^ had the best fit to this path diagram, and had 0.53 of unexplained variation. The best multiple regression result (Supplementary Information “Regressions”) had the genes *amoA* Archaea and Bacteria, pH and soil N as variables; when their coefficients were used in a linear regression with NO_3_^-^, the *R*^2^ was 0.93 and *P* < 0.001 (**Figure 2D**).

To explain N_2_O flux, a 3D bubble plot showed the relationships among water content (WC), NO_3_^-^ and *nosZ* gene abundance (**Figure 3C**, a linear-mixed model indicated *R*^2^ = 0.89, AIC = 141). The path diagram indicated that soil N, pH, NO_3_^-^, *nirS* and *nirK* and *nosZ* genes and WC were statically significant to explain N_2_O flux, and it presented 0.29 of unexplained variation in this model (**Figure 1E**). The *nosZ* gene abundance had the best fit to the path diagram, and had only 0.08 of unexplained variation. The best multiple regression result (Supplementary Information “Regressions”) had *nosZ*, WC and NO_3_^-^ as variables; when their coefficients were used in a linear regression with N_2_O, the *R*^2^ was 0.90 and *P* < 0.001 (**Figure 2E**).

### Methane Dynamics

Methane flux in forest soils and soybean fields varied from consumption to production, and was characterized by high spatial variation (**Table 1**; **Figure 2B**). On average, new soybean sites acted as a methane sink, while forest and old soybean soils had close to a neutral flux; pasture was clearly a methane source.

Abundances of methanogenic (*mcrA*) and methanotrophic (*pmo*A) genes were similar for each land use and were strongly correlated (0.82, *p* < 0.001); gene abundances were highest in forest sites, and lowest in soybean soils (**Table 1**). No direct correlations between abundances of these genes and methane fluxes were detected (Supplementary Table S4). Path diagram analysis indicated that soil pH, C, bulk density and the abundance of *mcrA* and *pmoA* genes were statically significant to explain CH_4_, and it presented 0.26 of unexplained variation in this model (**Figure 3D**). The best multiple regression result had soil C, pH, density and the *mcrA* gene as variables (Supplementary Information “Regressions”); when their coefficients were used in a linear regression with CH_4_, the *R*^2^ was 0.88 and *P* < 0.001 (**Figure 2B**).

### Linking Gene Abundances, Soil Parameters, and Processes

Gene abundances were correlated with soil chemistry and total C and N dynamics. The strongest correlations were obtained between genes and pedological factors involved in denitrification, namely NO_3_^-^, *nirK* and *nosZ* gene abundances, and N_2_O flux, but several other weaker correlations were also observed (Supplementary Table S4). Using path analysis the dependencies of these variables was tested in five models, namely microbial activity, ammonium mineralization, nitrification, denitrification, and methane dynamics (Supplementary Figure S1), and reduced models that present significant paths are shown (**Figure 1**). We also demonstrated the fit of these variables using multiple regressions, indicating that the marker genes fit well into the tested biogeochemical models (**Figure 2** and Supplementary Information “Regressions”).

For a better overview of the analyzed soil parameters (genes and chemistry) and their relationships by land use type, a PCA was performed (**Figure 4**). Each variable (soil parameter) is represented by a vector, and the length of each vector indicates the strength of its contribution. The relative importance of each variable can be estimated from the perpendicular projection of each sample to its respective vector. For example, C was highest in the forest samples (if one takes a perpendicular imaginary line to the C vector, one can see that forest samples are at the top of this vector). The two main axes (PC1 and PC2) indicate the total variance of the data explained in the PCA (58 and 16%, respectively). Thus, the forest soils were best characterized by high quantities of OM, CEC, C, N_2_O, N, NO_3_^-^, NH_4_^+^, and most of the marker genes. All soybean soils were characterized by high pH and soil fertility (P, Ca+Mg, and S), while soybean 2y had high soil CN and CO_2_ flux and the soybean 25y and pasture soils had high abundance of *amoA* Archaea and 16S Archaea. Pasture sites were characterized by high *nirS* abundance and CH_4_ flux. In addition to the PCA, n-MANOVA analysis was performed and showed that the discrimination of the land use types (as observed in the PCA by the different cluster of symbols) was statistically significant (*P* < 0.05).

**FIGURE 5:**
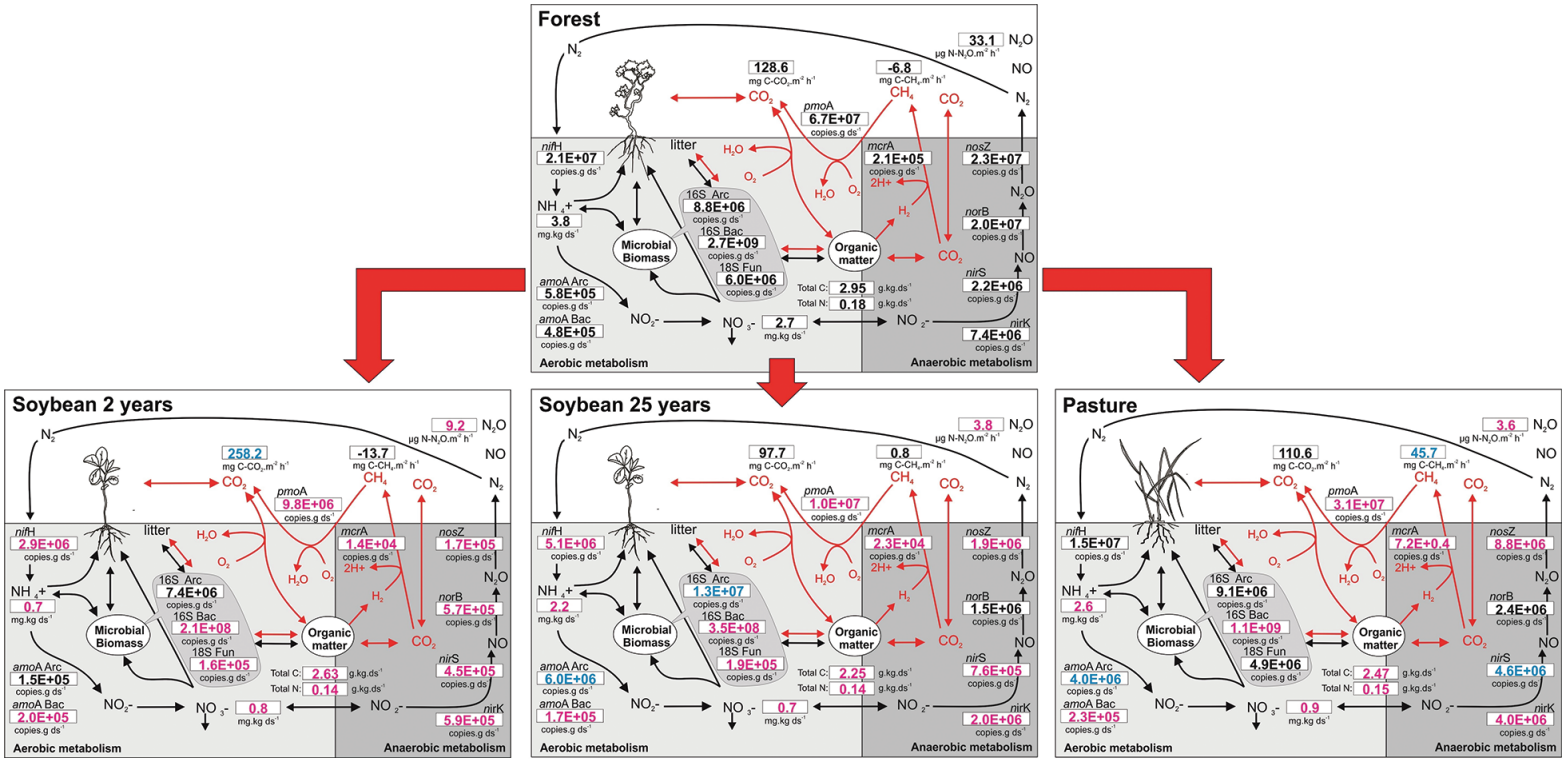
**Integrated scheme for the carbon and nitrogen cycles in response to land use change in the soils of Southern Amazonia.** The abundance of soil microbial genes involved in biogeochemical cycles of C and N are shown together with their respective end products. The forest was set as the reference value representing an unchanged control. Values that are modified with land use change from forest to soybean fields or pasture are indicated in color (increases in blue, and decreases in magenta; Tukey’s *post hoc* test, *P* < 0.05).

Finally, to facilitate the visualization of all the presented data (gene abundances and their chemical substrates and products) in the context of C and N cycles in soils, we propose an integrated diagram of soil chemical parameters, gene abundances, and gas fluxes for the effects of this land use change (**Figure 5**). Overall, land use affected the microbial groups, and gene abundance was a sensitive indicator that correlated positively with C and N forms and fit well into the tested biogeochemical models (**Figures 1–3**).

## Discussion

In this study, we showed that the effects of land use change on microbial functional groups, accessed by their marker genes, provided useful information to explain the reported variations in total N and GHG fluxes (for CO_2_, CH_4_, and N_2_O) in Southern Amazonia soils. While land use type, seasonal management, and soil chemistry affected microbial abundance, the abundance of specific marker genes for microbial functional groups linked to soil parameters using multivariate tools contributed to explained values of C- and N-related process rates (**Figures 1–3**).

### Microbial Abundance and Activity

The highest microbial abundances, obtained in forest sites, were correlated with the highest concentrations of C, N, NH_4_^+^, and NO_3_^-^ in the forest sites. These characteristics are often observed in pristine soils when compared with agricultural soils, and are explained by litter degradation and tight nutrient cycling (Brando et al., 2008; Wieder et al., 2013). A previous study indicated that these forest sites had a more extensive litter layer and a more distinct chemical composition compared to the agricultural sites (Lammel et al., 2015). Litter quantity and quality were different between forest and agricultural sites, with the natural vegetation presenting higher proportions of recalcitrant compounds and a higher C:N ratio, which supports higher C accumulation and constant N mineralization in forest soils compared with agricultural soils (Wieder et al., 2013; Lammel et al., 2015). Also, abundance of N-fixing microorganisms was highest in forest soils, which over a long time period can account for higher soil N levels (Morales et al., 2010).

Thus, we associate the highest microbial abundance in the forest sites during the wet season with the highest levels of soil C and N (**Figure 3**; Yao et al., 2000; Stevenson et al., 2014). Additionally, several aspects of agricultural management might alter the abundance of Bacteria and Fungi in soils, such as the chemical changes introduced by fertilization and pesticide application (Yao et al., 2000; Imfeld and Vuilleumier, 2012). A previous study in these areas reported agricultural management (plant cover, fertilization, and pesticides) as drivers for soil Bacterial community structures (Lammel et al., 2015). To manage plant diseases and control harmful insects and weeds during soybean growth, fungicides, insecticides, and herbicides are applied (Supplementary Table S2), which can all function as factors related to soil microbial suppression (Imfeld and Vuilleumier, 2012; Lammel et al., 2015). In our study, Archaea showed the highest abundance in old soybean soils, which is an intriguing result, as it would be expected that Bacteria would have higher abundance compared to Archaea in response to liming and fertilization (Bengtson et al., 2012; Tripathi et al., 2013). Since the opposite result was observed in the present study, we suggest that while pesticides likely suppressed Bacterial and Fungal abundance in soybean fields, other factors selected for Archaeal abundance. Archaeal abundance is reportedly variable in soils at pH values below 4.7 (such as our forest sites); the higher pH value in the soybean site, the lower C:N ratio, and increased soil compaction leading to anaerobic microsites, as observed after the conversion from forest to agriculture in our study, might be drivers for the abundance of this group (Bates et al., 2011; Bengtson et al., 2012; Karlsson et al., 2012; Šibanca et al., 2014). Plant cover might be important to Archaeal abundance, since root exudates can influence the abundance of this group in the rhizosphere and in the bulk soil. Differences in tillage practices might also have an impact on Archaeal abundance; new soybean 2y had low abundance of Archaea, perhaps because conventional till practices affect the O_2_ status of the soil, whereas the 25y established soybean plots under no-till might have selected for this group (Karlsson et al., 2012; Sugiyama and Yazaki, 2012). We are not able to explain Archaeal abundances with precision at this time and future studies should clarify this phenomenon.

Elevated CO_2_ fluxes indicated higher microbial activity in new soybean soils. These fields were managed using conventional tillage, which is used in the 1st years after deforestation to aid in the incorporation of liming and in the decomposition of forest roots. Microbial respiration is usually more intense under these conditions in response to elevated soil pH, oxygenation by plowing, and increased levels of bioavailable carbon from the forest roots and debris (Carvalho et al., 2007; Pes et al., 2011). The opposite is observed in no-till systems, such as the old soybean and pasture fields, where native vegetation and agricultural soils produced similar CO_2_ fluxes, as observed in the present study for all other land uses (Carvalho et al., 2010). These results show that not only direct correlations between genes and process rates should be investigated, but also the relationships between them and other environmental parameters. In this case both the path analysis and the multiple regressions detected the importance of C, pH and the abundance of Archaea and Bacteria in explaining the CO_2_ flux rates (**Figures 1** and **2**). However, soil management factors such as oxygenation by plowing (parameter not measured) should also be taken into consideration in explaining the observed results (Carvalho et al., 2007; Pes et al., 2011).

### N-Cycle Dynamics

Archaeal ammonium oxidizers were more abundant than AOB in all areas, and were most abundant in agricultural soils (Leininger et al., 2006; Meyer et al., 2013). Concurrently, AOA, similar to total Archaea, and opposing AOB, were most abundant in higher pH old soybean fields, and had lower abundance in the acidic forest soils. Since generalizations around the effect of pH are not universally applicable and should be taken with caution, we suggest that agricultural land use selected for higher abundance of AOA, as suggested by some studies (Prosser and Nicol, 2012; Jiang et al., 2014). Additionally, higher C (correlation C/AOB *R* = 0.55, *P* < 0.001) and NH_4_^+^ concentrations (correlation NH_4_^+^/AOB *R* = 0.59, *P* < 0.01) in forest sites might have selected for higher abundances of AOB in those soils than in the agricultural soils, since AOB are generally more sensitive to such drivers (Verhamme et al., 2011; Stempfhuber et al., 2014). Interestingly, AOA followed the Archaeal abundance and we are not able to explain in details at this time why old soybean fields selected for this group.

Although AOA were more abundant in all soils, AOB presented the best fit explaining NO_3_^-^ (**Figure 3B**), similar to a previous report for potential nitrification rates in a laboratory analysis (Petersen et al., 2012). However, it is difficult to quantify nitrification rates in the field as these rates depend on multiple factors, including N availability, AOA and AOB interactions, the plant influence on the microbial community, plant uptake of NH_4_^+^/NO_3_^-^, and other environmental factors. Xia et al. (2013) was recently able to identify a better correlation of nitrification with AOB by using stable isotopes in soils cultivated with soybeans. Future studies with stable isotope probing might better delineate the contributions of AOA and AOB to soil nitrification in tropical soils under land use change.

In contrast to the distribution of nitrifiers, denitrifiers (*nirK* and *nirS* genes) were more abundant in forest and pasture sites. Abundance of *nirK-* and *nirS*-type denitrifiers differed significantly between land uses. While *nirK*-type denitrifiers were distinctly dominant in forest sites, the ratio of *nirK*/*nirS* was high in forest and soybean soils. Variable abundance ratios of *nirK* and *nirS* genes have been reported, with a trend of *nirK*/*nirS* ratios usually >1 (Bárta et al., 2010; Enwall et al., 2010; Meyer et al., 2013; Jones et al., 2014). Little is known about the drivers for the differentiation in abundance of *nirK* or *nirS*-gene denitrifiers in soils, but elevated soil pH and Cu concentration have been reported as important factors having a positive influence on *nirK* abundance (Enwall et al., 2010; Bru et al., 2011). Neither of these characteristics was observed in our data. New soybean field soils contained the highest Cu content, however, *nirK* genes were most abundant in forest soil. Plant cover and soil chemistry might be similarly important in determining the distribution of denitrifiers in soil, i.e., specific plants might select for a higher abundance of *nirK*-gene bearing denitrifiers (Bremer et al., 2007; Meyer et al., 2013).

A strong correlation was observed between abundances of *nirK* and *nos*Z genes (0.92, *p* < 0.001). However, there was no correlation between the subtraction of *nos*Z from *nirK*/*nirS* copy numbers with N_2_O fluxes, as suggested by Morales et al. (2010). In fact, some bacteria that possess *nos*Z clade I (our primer target) also have the *nirK* or *nirS* genes (∼67% of the analyzed genomes by Jones et al., 2008). The recently discovered *nos*Z clade II (not covered by our primers) has also been shown be ubiquitous and important in soils, showing that *nirK*/*nirS* and *nosZ* ratios are much more complex than previously believed (Jones et al., 2014). Interestingly, our data agree with the laboratory study by Petersen et al. (2012), who found that *nosZ* clade I abundance is a good predictor of denitrification (**Figure 5** C1–3; correlation *nosZ*/N_2_O *R* = 0.61, *p* < 0.003). While Petersen et al. (2012) based their findings on potential rates, our study corroborated the concept of *nosZ* as a bioindicator of denitrification with actual gas flux rates measured *in situ* at the same soil sampling time (Rocca et al., 2014). This is an interesting finding, since N_2_O flux is a complex process involving other pathways, such as anammox (Butterbach-Bahl et al., 2013). Further studies should include other processes and soil types, to test why *nosZ* clade I is such a good indicator for denitrification in some soils (Rocca et al., 2014).

In this context, the term ‘denitrification regulatory phenotype’ (DRP) has been proposed for the denitrification process, which includes a series of analyses of a community, even though all the players and processes are not completely understood (Bergaust et al., 2011). For instance, it encompasses the most important traits of the microbial activity in the denitrification process in the environment (Bergaust et al., 2011). Using this approach the most important parameters are measured (i.e., pH, NO_3_^-^, O_2_, *nos*Z genes) and the overall results reported (i.e., N_2_O and NO_x_). This technique is particularly powerful for microcosm incubation, whereby soil samples can be transported from the field to the laboratory and incubated to measure the effect of O_2_ and NO_3_^-^ in the NO_x_ and N_2_O fluxes of that particular microbial community, allowing a better understanding of denitrification in that soil sample. In this study we worked with measurements *in situ* that made these evaluations hard to evaluate under field conditions, and we suggest that this approach could generate very informative data for future work that could better explain the insights observed in our data.

The highest N_2_O eﬄux from acidic forest soils and additional correlations with N concentrations, soil pH values, and total soil C were also observed in the present study (Richardson et al., 2009; Petersen et al., 2012). Paradoxically, even though land use change to agriculture affected the N-related microbial groups, it was beneficial in reducing potential nitrate leaching and N_2_O emissions, mitigating environmental pollution and global warming (Wieder et al., 2013).

### Methane Dynamics

Forest soils had the highest abundance of methanogens, however, pasture had the highest methane eﬄuxes, showing that rather than looking for direct correlations between microorganisms and process rates, they need to be included in a multivariate framework (**Figure 3**). In pasture soils, cow grazing compacts the soil, resulting in more anaerobic sites, and consequently higher methane production (Frey et al., 2011; Šibanca et al., 2014), as detected by both the path analysis and multiple regressions (**Figure 3**). In addition, cow excrement, which is more easily degradable than forest litter, might serve as a nutrient source for soil microbes leading to increased methanogenic activity and probably also supplying methanogens to the soil (Gattinger et al., 2007; Prem et al., 2014). Methane dynamics are influenced by diffusional constraints on the net oxidation activity, which would be important to consider in future studies (Striegl, 1993).

The quantity of methanotrophs, represented by detection of the gene *pmo*A, correlated with the distribution of methanogens (*R* = 0.82, *p* < 0.001). Since the methanotrophs potentially consume the methane produced by methanogens, it is a plausible explanation for most of our observed data, as demonstrated by the path analysis (**Figure 3**). Additionally, Forests are known as net methane sinks, and methanotrophs are usually associated with this vegetation system (Conrad, 2002; Cerri et al., 2006). By the other hand, in the crop fields fungicides and pesticides can suppress methanotroph communities (Conrad, 2002). In fact, the *pmoA* gene abundance was correlated with total Bacterial abundance and their mutual suppression in soybean fields in the wet season is very likely related to crop management (e.g., pesticides and fungicides). We observed that after winter fallow the methanotroph abundances were more similar among the land uses (Supplementary Figure S2), showing that specific agricultural management during soybean growth affected these communities and that abundance of methanotrophs could be nearly restored after winter fallow.

### Linking Gene Abundances, Soil Parameters, and Processes

Our data demonstrate that rather than searching for direct correlations between genes and biogeochemical processes, environmental factors, such as soil parameters, should also be included using regression analyses (**Figures 4** and **5**). Gene abundances have some correlation with processes, but the relationships of gene abundances with environmental factors are more important for analyzing the complex biogeochemical processes detailed here (Petersen et al., 2012; Levy-Booth et al., 2014). The net balance of chemicals in soil is a result of the interaction among the abundance and activities of microbial functional groups, the plants, and the soil physical and chemical characteristics (Levy-Booth et al., 2014). While climatic conditions, temperature and rain, can impact process rates within short time periods (hours–days) and soil characteristics (i.e., texture, total C) affect long-term predictions (seasons-years), gene abundances are important indicators for mid-term predictions (weeks–months) and for detailed microbial transformation dynamics and their effects on biogeochemical processes (Petersen et al., 2012; Levy-Booth et al., 2014).

Biogeochemical processes in soil are complex and our knowledge of the microbial players and controlling factors is constantly growing. New processes have been discovered in recent years (e.g., annamox) showing that our knowledge of the microorganisms and processes is still incomplete. In practice, no study to date has evaluated all the possible processes and variables in the C and N cycles, but instead focused on some of the main aspects (Petersen et al., 2012; Levy-Booth et al., 2014; Rocca et al., 2014). It follows that our paper does not attempt to evaluate all the possible aspects of the C and N cycles, but in studying some important aspects we bring advances to this research area.

For the first time we have detailed how the abundance of functional groups varied with land use change in soils of Southern Amazonia, and how these abundances are related to biogeochemical C and N cycles and GHG fluxes. We present integrated models of C and N cycles, showing gene abundances as key microbial indicators. Further studies with larger temporal and spatial surveys could help to explain how these parameters change through time and in different soils, and might also provide a database for predictive models. Also, improved primer design, additional marker genes (e.g., Jones et al., 2014; Levy-Booth et al., 2014), and expanded metadata for physico-chemical parameters, such as quantification of soil anaerobic micro-sites, and partitioning of SOM, could improve models and lead to a better understanding of the microbial regulation of biogeochemical processes in soils.

Metagenomic sequencing, which is primer independent, might be a more promising tool for true quantification of gene abundances in soil samples. However, cost is still a limiting factor. For example, while sequence analysis of a metagenomic library with minimum coverage costs around 2,000 dollars per sample, qPCR analysis costs around three dollars per gene within a sample (approximate prices accessed by the authors in 2014). Therefore, metagenomic sequencing can provide taxonomic and phylogenetic information, and thus both the vicinity of functional genes within other genomic content as well as material for better primer design. And qPCR is still a less expensive and powerful technique for gene abundance measurements, which allows integration of representative microbiological parameters into models of the C and N cycles. It enables broader surveys to analyze a higher number of samples, a requisite for modeling approaches.

## Conclusion

The abundance of microbial functional groups, as determined by appropriate marker genes, fit well into associated biogeochemical process models, indicating the value of these data for a better mechanistic understanding of biogeochemical cycles. Land use change affected the abundance of select microbial groups in soils. Overall, forest soil contained a higher abundance of all marker genes targeting processes in the soil C and N cycle compared to other land uses. A seasonal trend was observed for the marker gene abundance, suggesting that land use effects were less significant after the dry winter fallow and more intense during the wet season and the associated crop growth. This means that agricultural management caused a disturbance in the growth or survival of functional groups that was restored almost to native levels after the dry winter fallow. We showed that integrating gene abundance with environmental parameters presented a better overview of biogeochemical processes. Further temporal and spatial surveys of the parameters discussed might help to improve our understanding of the ecological regulation of biogeochemical cycles, and should be incorporated into future predictive models.

## Conflict of Interest Statement

The authors declare that the research was conducted in the absence of any commercial or financial relationships that could be construed as a potential conflict of interest.
