# Supplementary material for: Specific microbial gene abundances and soil parameters contribute to C, N, and greenhouse gas process rates after land use change in Southern Amazonian Soils
Source: Front Microbiol. 2015 Oct 6;6:1057. doi: 10.3389/fmicb.2015.01057 (PMC4594008; doi:10.3389/fmicb.2015.01057)
Supplement: Supplementary file 1 [file Data_Sheet_1.PDF]

frontiers

Supplementary Material

Specific microbial gene abundances and soil parameters contribute to C, N, and greenhouse gas process rates after land use change in Southern Amazonian Soils

Daniel R. Lammel[1,2], Brigitte Feigl[1], Carlos C. Cerri[1], Klaus Nüsslein[2*]

[1] Centro de Energia Nuclear na Agricultura, University of São Paulo, Piracicaba, SP, Brazil
[2] Department of Microbiology, University of Massachusetts, Amherst, MA, USA

*** Correspondence:** K. Nüsslein, Address: University of Massachusetts Amherst, Dep. of Microbiology, 203 Morrill Science Center IV-N, 639 North Pleasant Street, Amherst, MA, 01003-9298, USA
E-mail: nusslein@microbio.umass.edu

1. Supplementary Figures and Tables

1.1. Supplementary Tables

Supplementary Table 1. Quantitative gene detection for microbial target groups, the respective marker genes, and the related gene function, listed by primer sets and amplification conditions for the qPCR assays.

Microbial Groups	Marker Gene	Related Gene Function	Primers	Annealing Temp. (ºC)	References[1]
Archaea	*16S rRNA*	Ribosomal structure	915F/1059R	61	Yu et al., 2005
Bacteria	*16S rRNA*	Ribosomal structure	F563/BSR926	55	Claesson et al., 2010
Fungi	*18S rRNA*	Ribosomal structure	EF4/Fung 5r	59	Van Elsas et al., 2000
N-fixation	*nifH*	Nitrogenase enzyme	F/Rb	TD[1] 65-60	Rösch et al., 2005
Nitrification	*amoA* Archaea	Ammonia monooxygenase	F/R	63	Leininger et al., 2006
Nitrification	*amoA* Bacteria	Ammonia monooxygenase	1F/2R	TD 65-62	Rotthauwe et al., 1997
Denitrification	*nirK*	Cooper nitrite reductase	F1aCu/R3Cu	TD 65-60	Hallin et al., 1999
Denitrification	*nirS*	Iron nitrite reductase	Cd3aF/R3cd	TD 65-60	Throbäck et al., 2004
Denitrification	*cnorB*	Nitric oxide reductase	*cnor*BF/R	TD 65-62	Dandie et al., 2007
Denitrification	*nosZ*	Nitrous oxide reductase	2F/2R	TD 65-62	Henry et al., 2006
Methanogens	*mcrA*	Methyl coenzyme M reductase	Mlas/*mcr*AR	55	Steinberger et al., 2008
Methanotrophs	*pmoA*	Methane oxygenase	A189f/A661r	63	Holmes et al., 1995; Costello and Lidstrom, 1999

[1] For detailed references please see the main article; [2] TD, touchdown PCR.

Supplementary Table 2. Description of sampling sites with detailed land use history.

Characteristics	Sites Description		
Municipality	------------ Sinop -----------		Porto dos Gauchos
Site designation	Soybean 25y[1]	Pasture[2]	Soybean 2y
Year of Deforestation	1978	1988	2008
Former land uses	Guarana, Coffee	*Brachiaria humidicula*	Forest, Rice
Soybean or Pasture since:	1986	1988	2009
Recent land use history:			
2000/2001	Soybean	*Brachiaria brizantha*	Forest
2001/2002	Soybean	*B. brizantha*	Forest
2002/2003	Soybean	*B. brizantha*	Forest
2003/2004	Soy/Sorghum (DC)[1]	*B. brizantha*	Forest
2004/2005	Soy/Corn (DC)	*B. brizantha*	Forest
2005/2006	Soy/Sorghum (DC)	*B. brizantha*	Forest
2006/2007	Soy/Sorghum (DC)	*B. brizantha*	Forest
2007/2008	Soy/Millet (DC)	*B. brizantha*	Forest
2008/2009	Soy/Corn (DC)	*B. brizantha*	Rice
2009/2010	Soy/Corn (DC)	*B. brizantha*	Soybean
2010/2011	Soy/Corn (DC)	*B. brizantha*	Soybean

[1]DC, Double Cropping: during the summer, soybeans are cultivated first (usually October-February) followed by corn, millet, or sorghum (the seedling is made after the soybean harvest, usually in February-March). During the winter (dry season) the land is kept fallow. Soybean fields receive pesticides, which vary from year to year according to local recommendations. Common used products are: herbicides, glyphosate 2 l ha^{-1}; insecticides, metamidofos 200 ml ha^{-1}, permetrina 80ml ha^{-1}, aramo 200 ml ha^{-1}, flex 600ml ha^{-1}, and classic 40g ha^{-1}; and fungicides, opera (pyraclostrobin at 85 g/L and epoxiconazole at 62.5 g/L) 500 ml ha^{-1}, and carbendazin 500 ml ha^{-1}. Soybean annual fertilization is around of 250 kg per ha of N-P_2O_5-K_2O (00-20-20; triple superphosphate 33%, single superphosphate 33%, and KCl 34%, w/w), inoculation with *Bradyrhizobium* for N-fixation, and periodic liming (Ca and Mg carbonates) to elevate the soil pH to around 5.5. [2] Pastures rarely receive annual fertilization (usually single superphosphate) or liming.

Supplementary Table 3. Soil chemical and physical attributes in the surveyed areas.

Attribute	Unit	Forest		Soybean 2y		Soybean 25y		Pasture	
Soil chemistry									
pH		3.9	c[1]	5.1	a	5.0	a	4.6	b
C	$g.kg^{-1}$	2.95	a	2.63	b	2.25	d	2.47	c
N	$g.kg^{-1}$	0.18	a	0.14	b	0.14	b	0.15	b
OM [2]	$g.dm^{-3}$	45	a	39	b	36	b	38	b
CEC	$mmol_c.dm^{-3}$	91	a	80	b	68	c	69	c
K	$mmol_c.dm^{-3}$	0.8	b	0.9	b	1.3	a	0.8	b
Ca	$mmol_c.dm^{-3}$	2.0	c	25.0	a	27.7	a	11.7	b
Mg	$mmol_c.dm^{-3}$	2.0	c	19.7	a	6.3	b	5.7	b
P	$mg.dm^{-3}$	4.3	b	16.0	a	13.7	a	3.3	b
S	$mg.dm^{-3}$	9.0	b	23.3	a	13.3	b	9.7	b
B	$mg.dm^{-3}$	0.35	a	0.28	b	0.26	bc	0.23	c
Cu	$mg.dm^{-3}$	0.2	b	0.7	a	0.4	b	0.1	b
Fe	$mg.dm^{-3}$	273	a	94	b	59	c	118	b
Mn	$mg.dm^{-3}$	2.6	a	1.2	b	0.9	b	1.0	b
Zn	$mg.dm^{-3}$	0.3	b	2.2	a	1.9	a	0.4	b
Physical parameters									
Soil density	$kg.dm^{-3}$	0.93	b	1.04	ab	1.12	a	1.09	a
Soil Temp. (5 cm)	ºC	22.0	b	24.5	a	22.0	b	23.3	ab
Soil Temp. (10 cm)	ºC	21.4	b	23.5	a	22.0	ab	23.7	a

[1] Values with the same letter in one row are not different by Tukey's post-hoc test ($p<0.05$).
[2] OM, Organic Matter (determined by dichromate colorimetrically).
[3] CEC, cation exchange capacity.

Supplementary Table 4. Pearson correlation between soil parameters and concentrations of C and N, gene abundancies, and GHG fluxes. Only significant values are shown (R>0.4, p<0.1). In the triangle matrix, upper part represents *P* values and the lower part depicts R values.

	16S_Bac	16S_Arc	18S_Fung	*nif* H	*amo* A_Arc	*amo* A_Bac	*nor* B	*nir* K	*nir* S	*nos* Z	*mcr* A	*pmo* A	C	N	C:N	NH_4	NO_3	CH_4Flux	CO_2Flux	N_2OFlux	WC	Density	T5cm	T10cm	pH	OM	P	S	Ca+Mg	CEC	B	Cu	Fe	Mn	Zn
**16S_Bac**	1			<0.001		<0.001		<0.001		<0.001	0.002	<0.001	0.03	0.014			0.008			0.002		0.011			0.005	0.054	0.07		0.009	0.026	0.07		0.003	0.004	0.057
**16S_Arc**		1			<0.001																														0.067
**18S_Fung**			1	0.05	0.044	0.025		0.014	0.011	0.038	0.003	<0.001		0.05		0.042	0.01								0.011		0.011		0.01	0.054			0.035		0.015
***nif* H**	0.87		0.43	1		0.001		<0.001		<0.001	0.002	0.002		0.059			0.034			0.004		0.062			0.013		0.043		0.016	0.056			0.014	0.031	0.051
***amo* A_Arc**		0.69	-0.44		1	0.057				0.025		0.067	<0.001	<0.001			0.003			0.005		0.001			0.002	<0.001	0.018		0.015	<0.001	0.01		<0.001	<0.001	0.01
***amo* A_Bac**	0.69		0.49	0.66	-0.42	1		<0.001		<0.001	<0.001	<0.001	0.01	0.004		0.005	<0.001			0.056		<0.001			0.001	0.016			0.004	0.002	0.02		<0.001	<0.001	
***nor* B**							1																												
***nir* K**	0.87		0.53	0.82		0.73		1		<0.001	<0.001	<0.001	0.045	0.014		0.013	<0.001			0.061		0.004			<0.001	0.013	0.027		0.002	0.002	0.06		<0.001	0.004	0.018
***nir* S**			0.54						1			0.021															0.039								
***nos* Z**	0.91		0.45	0.80	-0.49	0.73		0.92		1	<0.001	<0.001	<0.001	<0.001		0.033	<0.001			0.003		<0.001			<0.001	0.005	0.007	0.071	<0.001	<0.001	0		<0.001	<0.001	0.004
***mcr* A**	0.63		0.62	0.64		0.67		0.77		0.76	1	<0.001	0.012	0.006		0.063	0.002			0.044		0.004			0.003	0.037	0.057		0.005	0.016	0.02		0.003	0.007	0.039
***pmo* A**	0.72		0.68	0.63	-0.41	0.74		0.87	0.50	0.85	0.82	1	0.004	0.001		0.006	<0.001					<0.001			<0.001	0.009	0.009		<0.001	0.001	0.02		<0.001	<0.001	0.007
**C**	0.48				-0.78	0.55		0.44		0.68	0.54	0.61	1	<0.001			<0.001	0.062		0.004		<0.001			<0.001	0.002	0.003		<0.001	<0.001	<0.001		<0.001	<0.001	0.002
**N**	0.53		0.43	0.42	-0.70	0.60		0.53		0.74	0.58	0.65	0.94	1		0.028	<0.001			0.002		<0.001			<0.001	0.002	0.001	0.063	<0.001	<0.001	<0.001		<0.001	<0.001	<0.001
**C:N**															1				<0.001				0.014					0.019							
**NH_4**			0.45			0.59		0.53		0.47	0.41	0.58		0.48		1	<0.001					0.002			<0.001	0.003	0.047		0.002	<0.001	0.06		0.002	0.018	0.018
**NO_3**	0.57		0.55	0.46	-0.61	0.72		0.77		0.79	0.64	0.83	0.69	0.75		0.74	1					<0.001		0.041	<0.001	<0.001	0.028		<0.001	<0.001	0		<0.001	<0.001	0.013
**CH_4Flux**													-0.41					1																	
**CO_2Flux**															0.78				1									0.002				0.039			
**N_2OFlux**	0.63			0.60	-0.59	0.42		0.42		0.61	0.44		0.60	0.64						1		0.002			0.01	0.009			0.039	0.015	0.01		0.005	0.001	
**WC**																					1														
**Density**	-0.55			-0.41	0.66	-0.67		-0.60		-0.75	-0.60	-0.70	-0.87	-0.93		-0.64	-0.86			-0.64		1		0.031	<0.001	<0.001	0.029		<0.001	<0.001	<0.001		<0.001	<0.001	0.007
**T5cm**															0.53								1	0.003											
**T10cm**																	-0.45					0.47	0.61	1							0.02			0.04	
**pH**	-0.59		-0.54	-0.53	0.62	-0.66		-0.67		-0.80	-0.61	-0.76	-0.83	-0.93		-0.67	-0.82			-0.55		0.9			1	0.001	<0.001	0.012	<0.001	<0.001	<0.001		<0.001	<0.001	<0.001
**OM**	0.43				-0.67	0.52		0.53		0.59	0.46	0.56	0.65	0.64		0.62	0.76			0.56		-0.78			-0.65	1			0.027	<0.001	0.04		<0.001	<0.001	0.022
**P**	-0.40		-0.54	-0.45	0.51			-0.48	-0.45	-0.57	-0.42	-0.56	-0.62	-0.66		-0.44	-0.48					0.48			0.78		1	0.005	<0.001		0.01	0.006	0.001	0.035	<0.001
**S**										-0.40				-0.41	0.51				0.62						0.54		0.59	1	<0.001		0.04	<0.001			0.002
**Ca+Mg**	-0.55		-0.55	-0.52	0.52	-0.60		-0.63		-0.75	-0.58	-0.73	-0.76	-0.87		-0.64	-0.73			-0.45		0.81			0.97	-0.48	0.85	0.67	1	0.003	<0.001	0.018	<0.001	<0.001	<0.001
**CEC**	0.48		0.43	0.42	-0.67	0.64		0.64		0.67	0.52	0.65	0.69	0.70		0.73	0.89			0.52		-0.85			-0.75	0.92			-0.61	1	0.02		<0.001	<0.001	0.03
**B**	0.41				-0.53	0.51		0.41		0.60	0.51	0.51	0.79	0.93		0.41	0.61			0.58		-0.82		-0.51	-0.83	0.45	-0.53	-0.45	-0.79	0.51	1		<0.001	<0.001	0.003
**Cu**																			0.45								0.58	0.88	0.51			1			0.01
**Fe**	0.62		0.46	0.53	-0.68	0.69		0.67		0.82	0.61	0.77	0.89	0.92		0.63	0.86			0.59		-0.95			-0.96	0.77	-0.66		-0.89	0.84	0.75		1	<0.001	<0.001
**Mn**	0.59			0.47	-0.68	0.68		0.60		0.78	0.57	0.69	0.89	0.92		0.51	0.83			0.66		-0.96		-0.45	-0.88	0.80	-0.46		-0.76	0.85	0.79		0.95	1	0.01
**Zn**	-0.42	0.41	-0.52	-0.43	0.55			-0.51		-0.60	-0.45	-0.57	-0.62	-0.72		-0.51	-0.53					0.57			0.82	-0.50	0.93	0.62	0.84	-0.48	-0.62	0.55	-0.71	-0.55	1

1.2. Supplementary Figures

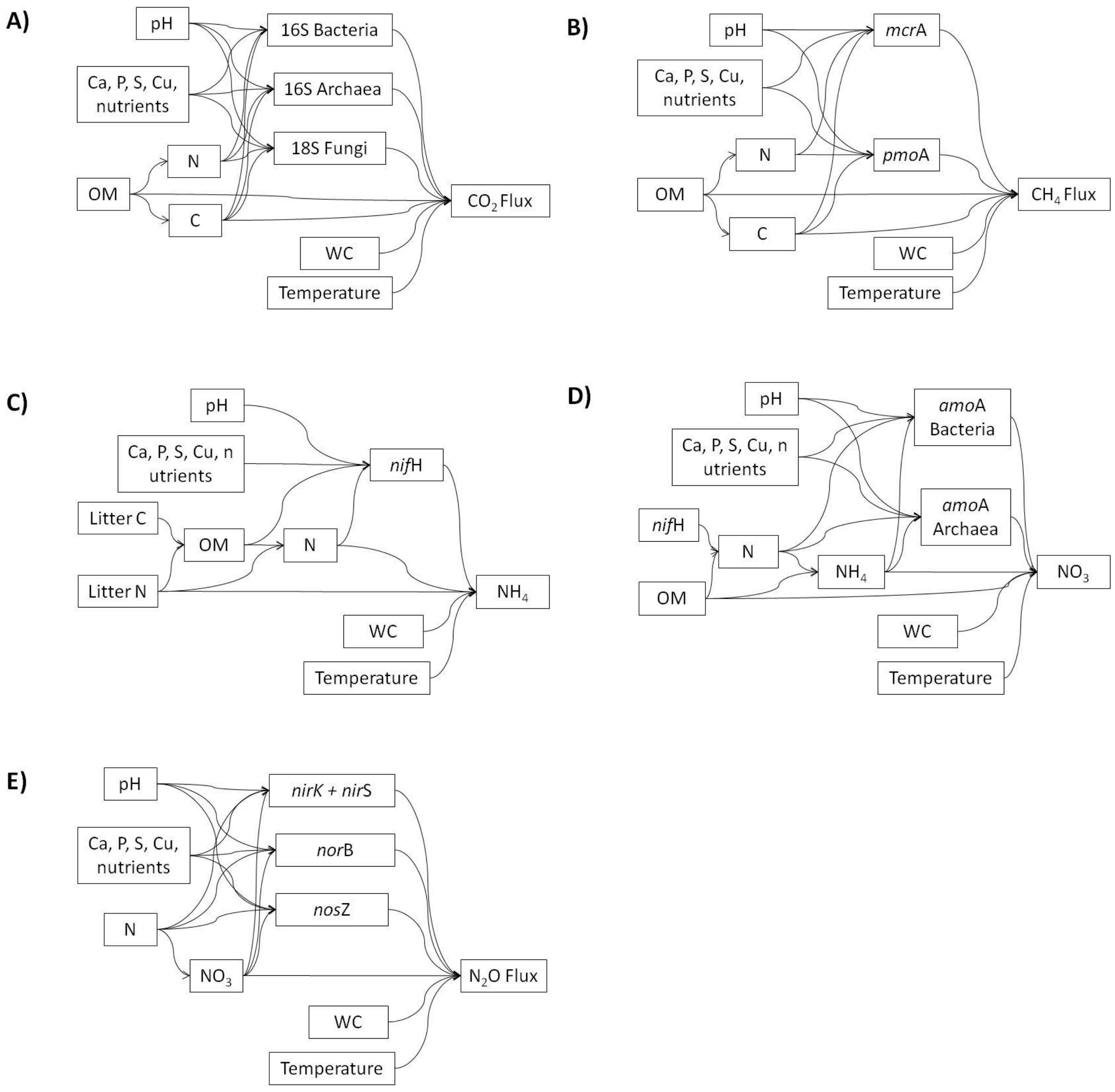

Supplementary Figure 1. Theoretical models tested with path analysis to describe the directed dependencies among the set of respective variables for , (A) microbial activity, (B) methane flux, (C) ammonium mineralization, (D) nitrification, and (E) denitrification.

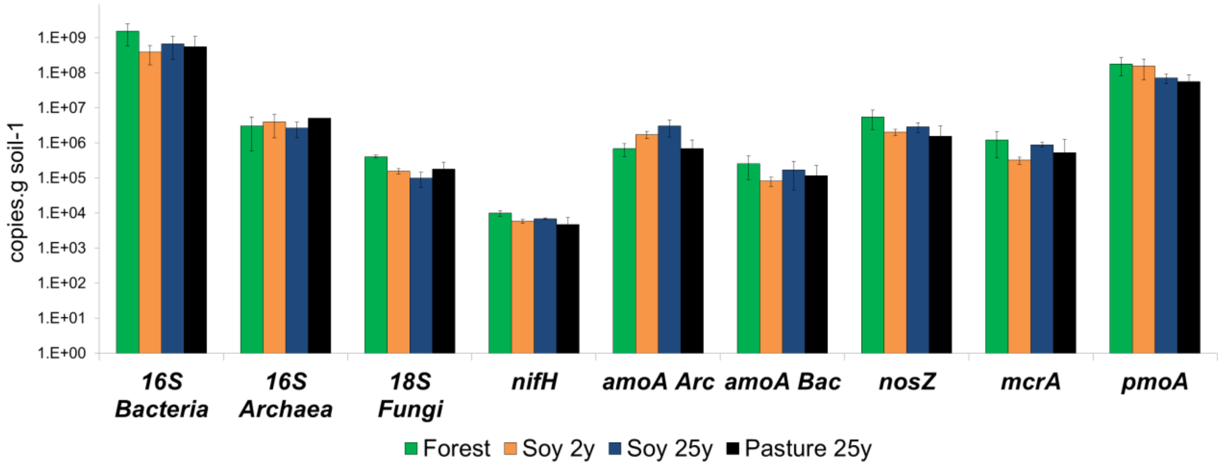

Supplementary Figure 2. Gene abundance for the different land uses at the onset of wet season (Nov. 2010), comparing pristine rainforest (For) with soybean fields two and twenty-five years after establishment (Soy 2y and Soy 25y), and with pasture (Pas).

2. Supplementary Information

Supplementary Regressions. Multiple regression analysis of selected variables related to CO_2, CH_4, NH_4, NO_3, and N_2O predictions, the type of regression (linear model, linear mixed effects model, and generalized additive model / fits semi and non-parametric data), and the respective fit value (R-squared, *P*-value, and/or AIC).

CO_2 Prediction

Linear model

```
lm(formula = CO2Flux ~ s16S_Bac + s16S_Arc + C + pH)

Residuals:
    Min      1Q  Median      3Q     Max
-38.100 -15.690  -3.173   6.674  75.922

Coefficients:
             Estimate Std. Error t value Pr(>|t|)
(Intercept) -1091.774    214.931  -5.080 0.000111 ***
s16S_Bac       17.546      9.249   1.897 0.076009 .
s16S_Arc      -16.144      8.051  -2.005 0.062167 .
C             212.899     45.513   4.678 0.000252 ***
pH            151.450     25.818   5.866 2.39e-05 ***
---
Signif. codes:  0 '***' 0.001 '**' 0.01 '*' 0.05 '.' 0.1

Residual standard error: 29.86 on 16 degrees of freedom
Multiple R-squared:  0.7115, Adjusted R-squared:  0.6393
F-statistic: 9.863 on 4 and 16 DF,  p-value: 0.0003214

 AIC 208.5436
```

Linear mixed-effect model

```
Fixed effects: CO2Flux ~ scale(X16S_Bac) + scale(X16S_ARC) + C + pH
                     Value Std.Error DF   t-value p-value
(Intercept)     -1082.8604 219.55544  7 -4.932059  0.0017
scale(X16S_Bac)    17.7609   9.33662  7  1.902280  0.0989
scale(X16S_ARC)   -16.1426   8.06203  7 -2.002296  0.0853
C                 210.5088  46.34486  7  4.542226  0.0027
pH                150.8082  26.45238  7  5.701119  0.0007
 Correlation:
                (Intr) s(X16S_B s(X16S_A C
scale(X16S_Bac) -0.164
scale(X16S_ARC) -0.095 -0.449
C               -0.924 -0.096    0.285
pH              -0.923  0.401   -0.110    0.708

Standardized Within-Group Residuals:
       Min         Q1        Med         Q3        Max
-1.2738051 -0.5326496 -0.0972199  0.2171189  2.4941199

> r.squared.lme(MReg_CO2)
  Class   Family     Link  Marginal Conditional      AIC
    lme gaussian identity 0.6586045    0.675818 212.5436
```

Generalized additive model

```
CO2Flux ~ s(s16S_Bac) + s(s16S_Arc) + C + pH

Parametric coefficients:
            Estimate Std. Error t value Pr(>|t|)
(Intercept)  -855.52     146.96  -5.821 0.000617 ***
C             218.73      29.86   7.326 0.000149 ***
pH             95.55      19.46   4.911 0.001664 **
---
Signif. codes:  0 '***' 0.001 '**' 0.01 '*' 0.05 '.' 0.1

Approximate significance of smooth terms:
              edf Ref.df     F p-value
s(s16S_Bac) 7.653  8.245 7.231  0.0065 **
s(s16S_Arc) 3.249  3.926 4.110  0.0490 *
---
Signif. codes:  0 '***' 0.001 '**' 0.01 '*' 0.05 '.' 0.1

R-sq.(adj) =  0.924   Deviance explained = 97.3%
GCV score = 552.96  Scale est. = 186.89    n = 21
AIC 176.4611
```

CH_4 Prediction

Linear mixed-effect model

```
Fixed effects: CH4Flux ~ s.mcrA + s.pmoA + pH + C + SoilDensity
                Value Std.Error DF   t-value p-value
(Intercept)  692.5347 135.00012 10  5.129882  0.0004
s.mcrA        13.8833   6.96999 10  1.991865  0.0744
s.pmoA       -15.5984   8.40429 10 -1.855999  0.0931
pH          -153.2430  30.24036 10 -5.067500  0.0005
C           -152.2904  26.71758 10 -5.700010  0.0002
SoilDensity  379.1737  99.54417 10  3.809100  0.0034
 Correlation:
            (Intr) s.mcrA s.pmoA pH     C
s.mcrA       0.171
s.pmoA      -0.383 -0.697
pH          -0.667 -0.216  0.403
C           -0.919 -0.189  0.190  0.601
SoilDensity  0.177  0.182 -0.152 -0.826 -0.246

Standardized Within-Group Residuals:
       Min         Q1        Med         Q3        Max
-1.0418418 -0.5361201 -0.2939574  0.3459286  2.3992850

> r.squared.lme(MNPReg)
  Class   Family     Link Marginal Conditional      AIC
    lme gaussian identity 0.662929    0.662929 190.7785
```

Generalized additive model 1

```
Formula:
CH4Flux ~ s(s.mcrA) + pH + C + SoilDensity

Parametric coefficients:
            Estimate Std. Error t value Pr(>|t|)
(Intercept)   773.11     111.26   6.949 2.06e-05 ***
pH           -165.01      24.85  -6.640 3.14e-05 ***
C            -172.99      22.40  -7.724 7.52e-06 ***
SoilDensity   403.16      80.00   5.040 0.000343 ***
---
Signif. codes:  0 '***' 0.001 '**' 0.01 '*' 0.05 '.' 0.1

Approximate significance of smooth terms:
            edf Ref.df    F p-value
s(s.mcrA) 5.643  6.505 3.17  0.0421 *
---
Signif. codes:  0 '***' 0.001 '**' 0.01 '*' 0.05 '.' 0.1

R-sq.(adj) =  0.799   Deviance explained = 88.6%
GCV score = 310.49  Scale est. = 167.92    n = 21
AIC 175.5662
```

Generalized additive model 2

```
CH4Flux ~ (s.mcrA) + s(s.pmoA) + pH + C + SoilDensity

Parametric coefficients:
            Estimate Std. Error t value Pr(>|t|)
(Intercept)   690.91     134.67   5.130 0.000123 ***
s.mcrA         13.88       6.97   1.992 0.064915 .
pH           -153.24      30.24  -5.068 0.000139 ***
C            -152.29      26.72  -5.700 4.21e-05 ***
SoilDensity   379.17      99.54   3.809 0.001711 **
---
Signif. codes:  0 '***' 0.001 '**' 0.01 '*' 0.05 '.' 0.1

Approximate significance of smooth terms:
          edf Ref.df     F p-value
s(s.pmoA)   1      1 3.445  0.0829 .
---
Signif. codes:  0 '***' 0.001 '**' 0.01 '*' 0.05 '.' 0.1

R-sq.(adj) =  0.632   Deviance explained = 72.4%
GCV score =  429.5  Scale est. = 306.78    n = 21
 AIC 186.7785
```

NH_4 Prediction

Linear model

```
la = NH4N ~ log(nifH) + OM + C + pH)

Residuals:
     Min       1Q   Median       3Q      Max
-1.65451 -0.65805 -0.03544  0.48881  2.75464

Coefficients:
            Estimate Std. Error t value Pr(>|t|)
(Intercept) 40.11280   10.78250   3.720 0.001861 **
log(nifH)   -0.43625    0.19338  -2.256 0.038427 *
OM           0.18776    0.07632   2.460 0.025639 *
C           -6.47353    1.73636  -3.728 0.001830 **
pH          -4.69582    1.00063  -4.693 0.000244 ***
---
Signif. codes:  0 '***' 0.001 '**' 0.01 '*' 0.05 '.' 0.1

Residual standard error: 1.068 on 16 degrees of freedom
Multiple R-squared:  0.7434,  Adjusted R-squared:  0.6792
F-statistic: 11.59 on 4 and 16 DF,  p-value: 0.0001307

 AIC 68.65626
```

Linear mixed-effect model

```
Fixed effects: NH4N ~ log(nifH) + OM + C + pH
                 Value Std.Error DF   t-value p-value
(Intercept) 12.693711 3.1189409 11  4.069879  0.0019
log(nifH)   -0.227130 0.0979931 11 -2.317820  0.0407
OM           0.468229 0.1666858 11  2.809050  0.0170
C           -3.553680 0.8490758 11 -4.185351  0.0015
pH          -1.273684 0.2550296 11 -4.994262  0.0004
 Correlation:
          (Intr) lg(nH) OM     C
log(nifH) -0.762
OM         0.060  0.129
C         -0.887  0.381 -0.176
pH        -0.775  0.499  0.312  0.756

Standardized Within-Group Residuals:
       Min         Q1        Med         Q3        Max
-1.4223108 -0.4822067  0.1275856  0.3243094  2.3427561

> r.squared.lme(MNPReg)
  Class   Family     Link  Marginal Conditional      AIC
    lme gaussian identity 0.7055516   0.7562915 45.34436
```

Generalized additive model

```
NH4N ~ s(log(nifH)) + OM + C + pH

Parametric coefficients:
            Estimate Std. Error t value Pr(>|t|)
(Intercept) 33.21912    9.05310   3.669 0.002072 **
OM           0.18776    0.07632   2.460 0.025639 *
C           -6.47353    1.73636  -3.728 0.001830 **
pH          -4.69582    1.00063  -4.693 0.000244 ***
---
Signif. codes:  0 '***' 0.001 '**' 0.01 '*' 0.05 '.' 0.1

Approximate significance of smooth terms:
             edf Ref.df     F p-value
s(log(nifH))   1      1 5.089  0.0382 *
---
Signif. codes:  0 '***' 0.001 '**' 0.01 '*' 0.05 '.' 0.1

R-sq.(adj) =  0.679   Deviance explained = 74.3%
GCV score = 1.4977  Scale est. = 1.1411    n = 21
 AIC 68.65626
```

NO_3 Prediction

Linear model

```
lm(formula = NO3N ~ log(amoA_Arc) + s.amoA_Bac + pH + N)

Residuals:
    Min      1Q  Median      3Q     Max
-0.8009 -0.5203 -0.1389  0.3711  1.3137

Coefficients:
              Estimate Std. Error t value Pr(>|t|)
(Intercept)     9.9093     8.5930   1.153   0.2658
log(amoA_Arc)  -0.1850     0.2534  -0.730   0.4760
s.amoA_Bac      0.3924     0.2030   1.933   0.0712 .
pH             -1.1850     0.7108  -1.667   0.1149
N              -2.5843    26.3335  -0.098   0.9230
---
Signif. codes:  0 '***' 0.001 '**' 0.01 '*' 0.05 '.' 0.1

Residual standard error: 0.6804 on 16 degrees of freedom
Multiple R-squared:  0.743,  Adjusted R-squared:  0.6787
F-statistic: 11.56 on 4 and 16 DF,  p-value: 0.0001322
AIC 49.71346
```

Linear mixed-effect model

```
Fixed effects: NO3N ~ log(amoA_Arc) + s.amoA_Bac + pH + N
                   Value Std.Error DF    t-value p-value
(Intercept)    15.398669  8.827329  7  1.7444313  0.1246
log(amoA_Arc)  -0.106811  0.263551  7 -0.4052775  0.6974
s.amoA_Bac      0.287308  0.194714  7  1.4755399  0.1836
pH             -1.954365  0.882601  7 -2.2143239  0.0624
N             -23.257703 26.866571  7 -0.8656744  0.4153
 Correlation:
              (Intr) l(A_A) s.mA_B pH
log(amoA_Arc) -0.607
s.amoA_Bac    -0.157 -0.164
pH            -0.684 -0.090  0.412
N             -0.927  0.470  0.090  0.583

Standardized Within-Group Residuals:
       Min         Q1        Med         Q3        Max
-1.1141595 -0.5700211 -0.1499116  0.2863230  1.9404100

> r.squared.lme(MNPReg)
  Class   Family     Link  Marginal Conditional      AIC
    lme gaussian identity 0.6097318   0.7617243 53.71346
```

Generalized additive model

```
NO3N ~ s(log(amoA_Arc)) + s(s.amoA_Bac) + pH + N

Parametric coefficients:
            Estimate Std. Error t value Pr(>|t|)
(Intercept)   4.9064     1.9011   2.581   0.0244 *
pH           -0.7789     0.4349  -1.791   0.0991 .
N             0.7453     0.4016   1.856   0.0888 .
---
Signif. codes:  0 '***' 0.001 '**' 0.01 '*' 0.05 '.' 0.1

Approximate significance of smooth terms:
                   edf Ref.df     F p-value
s(log(amoA_Arc)) 6.275  7.351 4.313  0.0123 *
s(s.amoA_Bac)    1.000  1.000 5.452  0.0379 *
---
Signif. codes:  0 '***' 0.001 '**' 0.01 '*' 0.05 '.' 0.1

R-sq.(adj) =  0.886   Deviance explained = 93.3%
GCV score = 0.29453  Scale est. = 0.1644     n = 21
AIC 29.99245
```

N_2O Prediction

Linear model

```
lm(formula = N2OFlux ~ WC + NO3N + scale(nosZ)

Residuals:
    Min      1Q  Median      3Q     Max
-23.904  -7.891   0.109   6.168  28.527

Coefficients:
             Estimate Std. Error t value Pr(>|t|)
(Intercept) -523.74891  220.21229  -2.378   0.0294 *
WC          2328.69652  938.15903   2.482   0.0238 *
NO3N           0.02548    4.28201   0.006   0.9953
scale(nosZ)   11.90909    4.82512   2.468   0.0245 *
---
Signif. codes:  0 '***' 0.001 '**' 0.01 '*' 0.05 '.' 0.1

Residual standard error: 12.72 on 17 degrees of freedom
Multiple R-squared:  0.5629, Adjusted R-squared:  0.4858
F-statistic: 7.299 on 3 and 17 DF,  p-value: 0.002356
AIC 171.9564
```

Linear mixed-effect model

```
Fixed effects: N2OFlux ~ WC + NO3N + scale(nosZ)
                Value Std.Error DF   t-value p-value
(Intercept) -367.8457  184.0511  8 -1.998606  0.0807
WC          1705.0280  780.0750  8  2.185723  0.0603
NO3N          -8.2527    4.3824  8 -1.883151  0.0964
scale(nosZ)   11.9478    4.1505  8  2.878649  0.0206
 Correlation:
            (Intr) WC     NO3N
WC          -0.999
NO3N        -0.499  0.472
scale(nosZ)  0.277 -0.260 -0.552

Standardized Within-Group Residuals:
        Min          Q1         Med          Q3         Max
-1.76425801 -0.48290227 -0.02429673  0.40003376  1.60816427

> r.squared.lme(MNPReg)#;VarCorr (MReg_N2O)
  Class   Family     Link  Marginal Conditional      AIC
    lme gaussian identity 0.2919823   0.7221559 175.0446
```

Generalized additive model

```
Formula:
N2OFlux ~ WC + s(NO3N) + s(nosZ)

Parametric coefficients:
            Estimate Std. Error t value Pr(>|t|)
(Intercept)   -428.8      161.5  -2.655   0.0285 *
WC            1918.8      697.0   2.753   0.0245 *
---
Signif. codes:  0 '***' 0.001 '**' 0.01 '*' 0.05 '.' 0.1

Approximate significance of smooth terms:
          edf Ref.df     F p-value
s(NO3N) 8.653  8.930 6.154 0.00688 **
s(nosZ) 2.190  2.503 5.064 0.03156 *
---
Signif. codes:  0 '***' 0.001 '**' 0.01 '*' 0.05 '.' 0.1

R-sq.(adj) =  0.892   Deviance explained = 95.6%
GCV score = 87.729  Scale est. = 34.078    n = 21
AIC141.5246
```
